# TANK Promotes Pressure Overload Induced Cardiac Hypertrophy *via* Activating AKT Signaling Pathway

**DOI:** 10.3389/fcvm.2021.687540

**Published:** 2021-09-03

**Authors:** Yanan Pang, Minglu Ma, Dong Wang, Xun Li, Li Jiang

**Affiliations:** ^1^Division of Cardiology, TongRen Hospital, Shanghai Jiao Tong University School of Medicine, Shanghai, China; ^2^Department of Cardiology, The First Affliated Hospital of Soochow University, Suzhou, China

**Keywords:** TRAF family member associated NF-κB activator, AKT signal pathway, scaffold protein, pathological cardiac hypertrophy, tansgenic mice

## Abstract

**Background:** TANK (TRAF family member associated NF-κB activator) acts as a member of scaffold proteins participated in the development of multiple diseases. However, its function in process of cardiac hypertrophy is still unknown.

**Methods and Results:** In this study, we observed an increased expression of TANK in murine hypertrophic hearts after aortic banding, suggesting that TANK may be involved in the pathogenesis of cardiac hypertrophy. We generated cardiac-specific TANK knockout mice, and subsequently subjected to aortic banding for 4–8 weeks. TANK knockout mice showed attenuated cardiac hypertrophy and dysfunction compared to the control group. In contrast, cardiac-specific TANK transgenic mice showed opposite signs. Consistently, *in vitro* experiments revealed that TANK knockdown decreased the cell size and expression of hypertrophic markers. Mechanistically, AKT signaling was inhibited in TANK knockout mice, but activated in TANK transgenic mice after aortic banding. Blocking AKT signaling with a pharmacological AKT inhibitor alleviated the cardiac hypertrophy and dysfunction in TANK transgenic mice.

**Conclusions:** Collectively, we identified TANK accelerates the progression of pathological cardiac hypertrophy and is a potential therapeutic target.

## Introduction

With the aging of population, heart failure, as the end stage of various overload cardiomyopathies has become a worldwide public health problem (1). Hypertensive cardiomyopathies cause elevated blood pressure in the left ventricular wall, which triggers cardiac hypertrophy as an adaptive response (2). However, prolonged hypertrophy progresses to multifaceted pathological changes: cardiomyocyte enlargement, myofibrillar assembly, fibrosis accumulation, and expression of a set of genes that discriminate hypertrophic growth from normal growth (3, 4). In recent decades, numerous parallel effectors in signaling transduction have been reported to be involved in the development of pathological cardiac hypertrophy (5).

The TNF receptor associated factor (TRAF) family member associated NF-κB activator (TANK) was first identified in 1996 (6) and is also known as TRAF-interacting protein (I-TRAF). It binds to all reported TRAF members except TRAF4 (7, 8). TANK exhibited both stimulatory and inhibitory properties at different expression levels during TRAF2-mediated NF-κB activation (9). The binding of TANK to TRAF3 promotes the phosphorylation of IRF-3 and IRF7, which is critical for the production of type 1 IFN in response to the recognition of viruses via TOLL-like receptors (TLRs) and acid-inducible gene-1 (RIG-1) (10, 11). Furthermore, TANK takes part in ubiquitination via regulating TRAF6, which acts as a ubiquitin ligase. Upon stimulation of the receptor activator of NF-κB (RANK) ligand (RANKL), markedly increased osteoclastogenesis in TANK-null cells was observed, with elevated ubiquitination of TRAF6 and activation of NF-κB (12). Apart from the interaction of TRAFs, TANK associates with the IKK-related kinases TANK binding-kinase 1 (TBK1) and IKKε, which functions as a scaffold protein (13). Several members of the TRAF family have been implicated in the development of cardiac hypertrophy, including TRAF3, TRAF5, and TRAF6 (14–16). Previous studies have also revealed that the knockout of IKKε in mice accelerates cardiac hypertrophy via activating the AKT and NF-κB signaling pathway (17). The overexpression of SIKE (suppressor of IKKε) attenuated cardiac hypertrophy by regulating the TBK1-AKT signaling pathway (18). However, the role of TANK in pathological cardiac hypertrophy has not yet been clarified.

In this study, we determined the expression of TANK in hypertrophic hearts and elucidated the potential signaling transduction pathway regulated by TANK. TANK was significantly upregulated in murine hearts subjected to aortic banding (AB). A cardiac-specific TANK transgenic (TANK-TG) mouse model showed accelerated pressure overload-induced cardiac remolding while the deletion of TANK exhibited a protective effect on cardiac hypertrophy and fibrosis. Mechanistically, TANK was involved in the activation of AKT, a central hypertrophic signaling effector. These data suggest TANK is a candidate for regulating pathological cardiac hypertrophy in response to sustained hemodynamic overload.

## Materials and Methods

All animal protocols were approved by the Animal Care and Use Committee of TongRen Hospital, Shanghai Jiao Tong University School of Medicine (No.2018-015).

### Reagents

Detailed information regarding the reagents used can be found in Table 1.

**Table 1:** Information of reagents used in experiment.

**Antibody**	**Manufacturer**	**Catalog number**	**Source of species**	**Dilution**
TANK	CST	2141	Rabbit	1:1,000
ANP	Abclonal	A1609	Rabbit	1:1,000
β-MHC	Proteintech	22280-1-AP	Rabbit	1:1,000
p-MEK	CST	9154	rabbit	1:1,000
MEK	CST	9122	rabbit	1:1,000
p-ERK	CST	4370	rabbit	1:1,000
ERK	CST	4695	rabbit	1:1,000
p-JNK	CST	4668	rabbit	1:1,000
JNK	CST	9252	rabbit	1:1,000
p-p38	CST	4511	rabbit	1:1,000
p38	CST	9212	rabbit	1:1,000
p-AKT	CST	4060	rabbit	1:1,000
AKT	CST	4691	rabbit	1:1,000
p-mTOR	CST	2971	rabbit	1:1,000
mTOR	CST	2983	rabbit	1:1,000
p-GSK3β	CST	9322	rabbit	1:1,000
GSK3β	CST	9315	rabbit	1:1,000
p-p70S6K	CST	9208	rabbit	1:1,000
p70S6K	CST	2708	rabbit	1:1,000
GAPDH	CST	2118	rabbit	1:1,000
TGFβ1	CST	3709	rabbit	1:1,000
p-Smad2	CST	3108	rabbit	1:1,000
Smad2	CST	3103	rabbit	1:1,000
p-Smad3	CST	9520	rabbit	1:1,000
Smad3	CST	9513	rabbit	1:1,000
Flag	MB	M185	mouse	1:2,000
HA	MBL	M180-3	mouse	1:2,000

### Cardiac-Specific TANK Knockout Mice

Cardiac-specific TANK mice (TANK-CKO) were generated by utilizing a Cre-loxP system. First, the locations of two single-guide RNAs (sgRNA) that flanked exon 3 of the TANK gene were designed using an online CRISPR Design Tool. The target sequence of each sgRNA was sgRNA1 (AAAAATAGTGTCAAACTGTTGAC-TGG) and sgRNA2 (GCAGGGTTTCTCTGTTATAGCCC-TGG), respectively, and was transcribed using a MEGAshortscript™ Kit (AM1354, Ambion). A T7 mMESSAGE mMACHINE Kit (AM1345, Ambion) was used to transcribe the Cas9 plasmid (pST1374-NLS-flag-linker-Cas9, Addgene, 44758). Then, both Cas9 mRNA and sgRNAs were purified using an miRNeasy Micro Kit (Qiagen, 217084). Exon3 was inserted into a backbone vector pBluescript SK(+)-2loxP flanked by two mloxP sites and two homology arms as a donor vector. The donor vector was purified using the QIAquick Gel Extraction Kit (Qiagen, 28704), then the mixture which contains the Cas9 mRNA and sgRNAs (10 ng/ul) along with donor vector (2.0 ng/ul) were injected into zygotes by utilizing a microinjection system (FemtoJet 5247). The genomic DNA of mice was extracted and detected to identify founder mice that contained floxed exon3 on the same allele. The following primers were used to confirm that the two loxPs were on the same allele: TANK-loxp-NF1: GGTTTCTTCACGGAAGTTGG; TANK-loxp-NR2: GCAAGTTGCCTACTTATTGAGTTCT. After F1 offspring were obtained, heterozygotes were screened by PCR using the following primers: TANK-loxP-NF3: TTGTAGGAAATGAGGAAGTGGA, TANK-loxP-NR2: GCAAGTTGCCTACTTATTGAGTTCT. Homozygous TANK-flox mice were generated from mating between heterozygotes using the same screening technique. Flox mice are born according to the genetic laws of Mendel. Then, the TANK^Flox/Flox^*-*α*-MHC-*MerCreMer mice were obtained by mating of TANK-Flox mice with α-MHC-MerCreMer (α-MHC-MCM) transgenic mice (The Jackson Laboratory, stock No. 005650). After 6 weeks, cardiac-specific TANK conditional knockout mice were established by an intraperitoneal injection of tamoxifen (25 mg/kg/day, Sigma, T-5648) for five consecutive days. The control groups (α-MHC-MCM mice and TANK-Flox mice) were treated with equal doses of tamoxifen injection.

### Cardiac-Specific TANK Transgenic Mice

First, we got the full-length cDNA of TANK gene from total RNA of mice by PCR. Then, the cDNA gene was cloned into the Bgl II and Hind III sites of pCAG-loxP-CAT-loxP-lacZ for expression. The vector was linearized by Sal I and purified like the donor vector described above. Subsequently microinjected into embryos (2.0 ng/ul) to generate the conditional transgenic mice. After collecting tail tissue of the 10-day offspring, founder mice were identified using DNA amplification by PCR: pcag-seq-F: CATGTCTGGATCGATCCCCG; Tank-seq-R: TCCAGAAGAAACTTCTTGTCG. CAG-loxP-CAT-loxP-TANK/α-MHC-MCM mice were generated by crossing with α-MHC-MCM transgenic mice. Finally, conditional TANK transgenic (TANK-TG) mice were obtained after injecting with tamoxifen intraperitoneally for five consecutive days. Next, a western blot (WB) was used to evaluate the expression of TANK. α-MHC-MCM mice were used as non-transgenic (NTG) groups with the same drug regimen.

### Animal Surgery

To induce cardiac hypertrophy, the mice underwent thoracic aortic banding (AB) surgery, as mentioned below (14). After being anesthetized using sodium pentobarbital via an intraperitoneal injection (80 mg/kg), the left chest of the male mice was opened to expose the thoracic aorta. Subsequently, ~70% aortic constriction was made with a specific needle tied around the thoracic aorta using a 7-0 silk suture. Sham-operated animals underwent every step without aorta ligation.

### AngII Induced Cardiac Hypertrophy

We conducted the mouse model of cardiac hypertrophy induced by Ang II infusion as previously described (16). Ang II (1.4 mgkg^−1^ per day and dissolved in 0.9% NaCl) was subcutaneously infused for 4 weeks using an osmotic minipump (Alzet model 2004, Alza Corp) implanted into each mouse. The control mice group were received the same procedures as the experimental animals, with the same dose of saline infusion.

### Echocardiography Assessment

A MyLab 30CV ultrasound system (Biosound Esaote Inc.) was used to perform echocardiography. The indicators were acquired from at least three consecutive cardiac cycles to evaluate cardiac function, including LV end-diastolic diameter (LVEDd), LV posterior wall thicknesses in diastole (LVPWd), LV end-systolic dimension (LVESd), end-diastolic interventricular septum diameter (IVSd), and fractional shortening (FS%). Calculation formula is FS(%) = (LVEDd-LVESd)/LVEDd ×100%.

### Histological Analysis

Hearts from the experimental animals of each group 4 weeks after operation were harvested and fixed in 10% formalin and embedded in paraffin. The samples were cut into sections of about 5 μm transversely. Hematoxylin and eosin (H&E) staining was used to calculate the myocyte cross-sectional area, and the collagen volume was assessed through picrosirius red (PSR) staining.

### Cardiomyocyte Culture and Recombinant Vectors

Isolating neonatal rat cardiomyocytes (NRCMs) from the hearts of 1–2-day-old SD rats was performed as described previously (19). Hearts excised from newborn SD rats were cut into pieces and digested using 0.03% trypsin 0.04% collagenase type II. NRCMs were harvested and grown in DMEM/F12 medium (C11330, Gibco) with 5-bromodeoxyuridine (0.1 mM) which inhibited fibroblast proliferation, penicillin/streptomycin, and 10% fetal calf serum (FCS) for 48 h. Subsequently, the NRCMs were maintained under serum-free conditions for another 12 h. To generate TANK-overexpressing stable clones, the TANK gene was transfected into a replication-defective adenoviral vector. Cardiomyocytes infected with vectors expressing GFP were used as controls. Consistently, a replication-defective adenoviral vector with Short hairpin RNA against TANK was used to knockdown TANK. Meanwhile, AdshRNA served as a control. Finally, cells infected with adenoviruses were grown in the aforementioned medium for 24 h, and were then incubated with PBS or angiotensin II (Ang II, 1 μmol/L) for an additional 24–48 h. Adenoviruses for infection were used at a multiplicity of infection (MOI) of 100 particles/cell for 24 h.

### *In vivo* and *in vitro* Inhibition Experiment

After AB, the solution containing LY294002 (L9908, Sigma), which is an PI3K inhibitor, was administered through an intraperitoneal injection at a dose of 50 mg/kg for 4 weeks. Meanwhile, the control groups were treated with a DMSO vehicle injection at the same volume. Transfected NRCMs treated with AKT inhibitor MK-2206 (MCE, 1 μmol/L) *in vitro* along with Ang II stimulation.

### Quantitative Real-Time RT-PCR and Western Blot

In brief, mRNA was extracted using TRIzol reagent, and cDNA was synthesized by reverse transcription from RNA. Quantitative real-time PCR was performed to detect the expression of selected genes with SYBR Green (Roche). GAPDH was used as the reference gene. Protein was extracted from ventricular tissues and cardiomyocytes using RIPA lysis buffer. A BCA Protein Assay Kit (Pierce) was used to determine protein concentration. After being separated through SDS-PAGE, proteins were transferred onto PVDF membranes and incubated with primary antibodies overnight at 4°C. After the secondary antibodies were added the next day, bands were visualized using an Odyssey Imaging System (LI-COR Biosciences). The levels of specific protein were determined by standardizing with the level of GAPDH on the same PVDF membrane. Primer information could be found in Supplementary Table 1.

### Immunoprecipitation

HEK293T cells were harvested after transfection for 24–48 h and lysed with IP buffer which consisted of 20 mM Tris-HCl (pH 7.4), 150 mM NaCl, 1 mM EDTA and 0.5% NP-40 and supplemented with a protease inhibitor cocktail (#04693132001, Roche). After incubated on ice for 20 min and centrifuged 13,000 g for 15 min, the cell lysate was obtained as the supernatant. Then, rabbit immunoglobulin G protein and A/G-agarose beads were added to the lysate and incubated at 4°C for 3 h. 500 μl cell lysate was incubated with 1 micro gram of antibody and 10 ml of protein A/G-agarose beads with gentle rocking at 4°C overnight. The precipitates were washed and acquired then subjected to WB with appropriate antibodies. Endogenous immunoprecipitation of TANK and AKT in TANK overexpressed NRCMs was performed similarly using indicated antibodies.

### Construction and Transfection of Plasmids

Human TANK and AKT overexpressed plasmids were constructed first. The primers were designed and full-length CDS sequences of TANK and AKT were amplified from homo cDNA. The full-length CDS sequences of TANK and AKT were inserted into pcDNA5-Fag-vector and pcDNA5-HA using In-fusion method, respectively. Primer sequences are as follows: AKT-S:TCGGGTTTAAACGGATCCATGAGCGACGTGGCTATTGTG; AKT-AS:GGGCCCTCTAGACTCGAGTCAGGCCGTGCCGCTG; TANK-S:TCGGGTTTAAACGGATCCATGGATAAAAACATTGGCGAGC; TANK-AS:GGGCCCTCTAGACTCGAGTTAAGTCTCTCCATTGAAGTGTGAATTAAG. The constructed recombinant plasmid was transfected into 293T cells with the assistance of transfection reagent PEI. After 24 h, the cells were collected and lysed on ice with IP buffer. The supernatant proteins were removed after centrifugation, 1 μg antibody and beads were added were incubated at 4° for 3 h. Wash the beads using 150-mm and 300 mM NaCl Buffers. Finally, 20–30 μl 2 × Loading Buffer was added to beads and boiled at 95°C for 10 min.

### Immunofluorescence Analysis

Immunofluorescence staining was performed to determine the surface area of the cell. After being infected with the indicated adenovirus for 24 h, PBS or Ang II (1 μmol/L) were used to stimulate NRCMs for 48 h and were finally fixed with 3.7% formaldehyde. NRCMs were immunostained with an α-actinin antibody (1:100 dilution) first, then were stained with a fluorescent secondary antibody (1:200). Image-Pro Plus 6.0 software was used to measure the surface area of the cell.

### Statistical Analysis

The values are represented as the mean ± SD. Comparisons between groups were performed using a two-tailed Student's *t*-test (two groups) or one-way analysis of variance (ANOVA) followed by Tukey's *post-hoc* test (more than two groups). A value of *P* < 0.05 was considered to suggest a statistically significant difference.

## Results

### TANK Expression Is Increased in Failing Murine Hypertrophic Hearts and Cardiomyocytes

**Figure 1:**
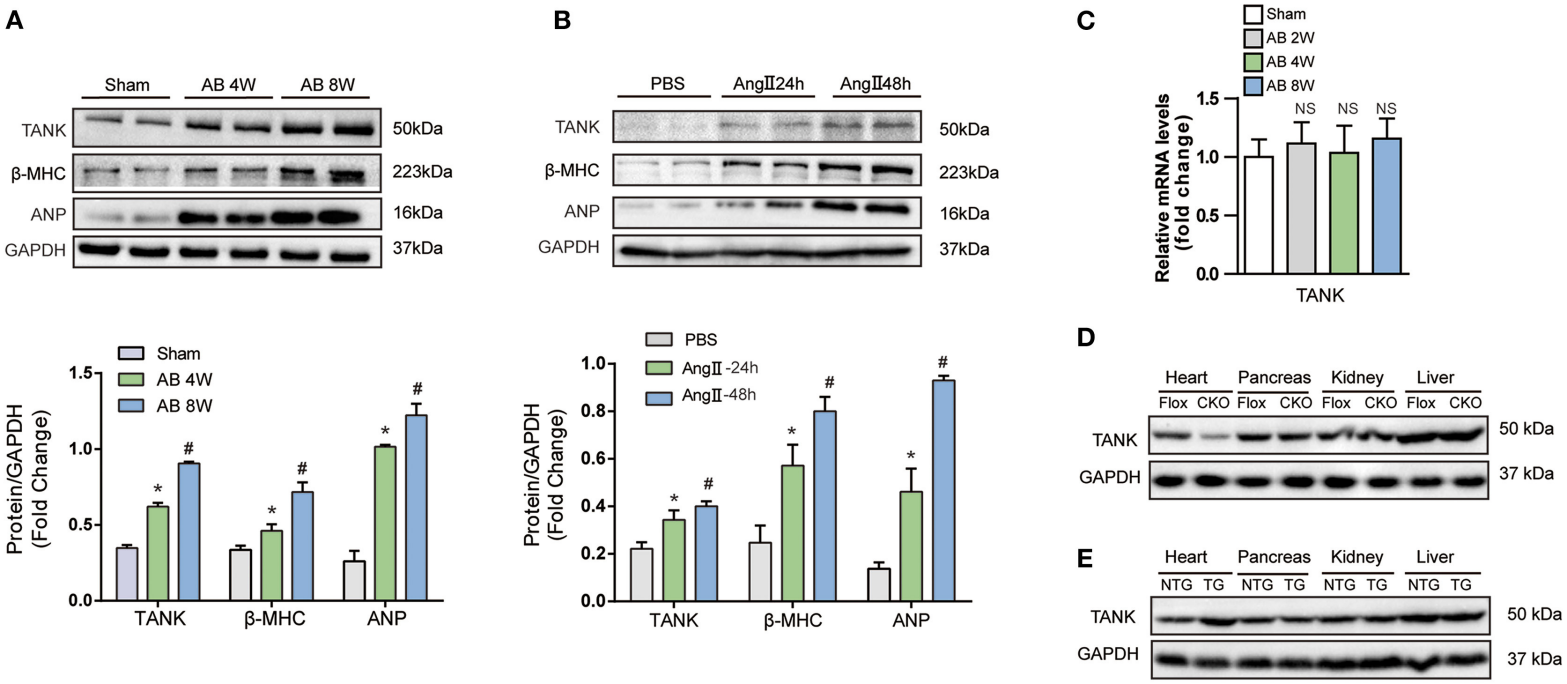
The expression of TANK is upregulated in hypertrophic murine hearts. **(A)** Western blot analysis of TANK expression in cardiac extracts of samples from mice subjected to aortic banding at the indicated time points (*n* = 6 mice per experimental group). **(B)** TANK expression was analyzed by western blotting in extracts from neonatal rat cardiomyocytes treated with angiotensin II (Ang II; 1 μmol/L) for 24 and 48 h (*n* = 3 independent experiments). ^*^P < 0.05 vs. Sham or vs. PBS; ^#^*P* < 0.05 vs. AB 2w or vs. Ang II 24 h. The data are presented as the mean ± SD. **(C)** mRNA levels of the TANK in heart samples from sham group and mice after aortic banding 2weeks, 4weeks and 8weeks (*n* = 3 per mice group). **(D)** Western blot showing TANK expression in TANK specific deleted heart, pancreas, kidney and liver. **(E)** TANK protein expression in heart samples and other tissue from TANK transgenic mice.

First, cardiac hypertrophy mouse models were established after aortic banding for 4 and 8 weeks to determine whether TANK is involved in pathological cardiac hypertrophy. WB was used to examine the expression of TANK and hypertrophic markers, including atrial natriuretic peptide (ANP) and β-myosin heavy chain (β-MHC). As shown in Figure 1A, the levels of TANK and hypertrophic markers were markedly elevated compared to the control group. During the development of cardiac hypertrophy, the expression levels of TANK, ANP, and β-MHC were more pronounced in 8 weeks than in 4 weeks. Similar results have been observed for *in vitro* experiments. Twenty-four or fourty-eight hours after angiotensin II administration, the expression of TANK and the hypertrophic markers was upregulated in NRCMs (Figure 1B). These results suggest that enhanced expression of TANK is related to the pathogenesis of cardiac hypertrophy. Quantitative RT-PCR was used to quantify the level of TANK mRNA in the heart samples of Sham group and AB group at 2, 4, and 8 weeks. As shown in Figure 1C, the expression of TANK mRNA had no change among all groups. Besides, we performed Western blot showing TANK expression in heart, pancreas, kidney, and liver samples of cardiac-specific TANK knockout mice (TANK-CKO) and TANK transgenic mice (TANK-TG) (Figures 1D,E).

### TANK Promotes AngII-Induced Cardiomyocyte Hypertrophy *in vivo* and *vitro*

**Figure 2:**
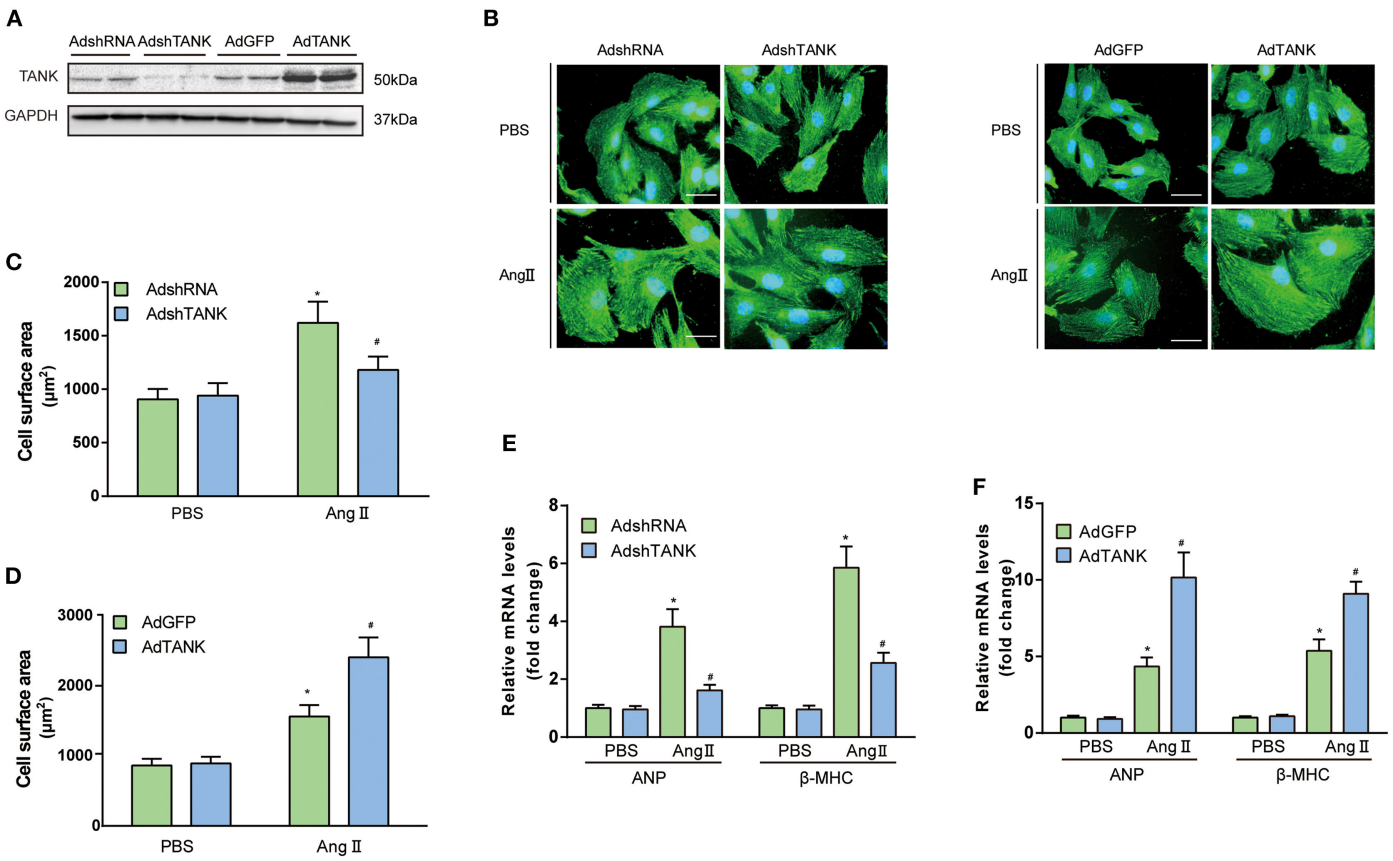
TANK augments the Ang II-induced hypertrophic response of cardiomyocytes. **(A)** Representative Western blot showing TANK expression in neonatal rat cardiomyocytes (NRCMs) infected with AdshRNA, AdshTANK, AdGFP, and AdTANK. **(B)** NRCMs were infected with AdshTANK or AdTANK (AdshRNA and AdGFP as control) and treated with Ang II (1 μmol/L) or PBS for 48 h. Sarcomere organization was stained using anti-α-actinin antibodies (green), and nuclei were determined by DAPI staining (blue); scale bar, 20 μm. **(C,D)** Quantification of the cell surface area in AdshRNA and AdshTANK **(C)** or AdGFP and AdTANK **(D)** treated with PBS or Ang II (*n* > 50 cells per experimental group; ^*^P < 0.05 vs. AdshRNA/PBS or vs. AdGFP/PBS; ^#^*P* < 0.05 vs., AdshRNA/Ang II or vs., AdGFP/ Ang II). **(E ,F)** Real-time polymerase chain reaction (PCR) analysis of hypertrophic markers atrial natriuretic peptide (ANP) and β-myosin heavy chain (β-MHC) in AdshRNA and AdshTANK **(E)** or AdGFP and AdTANK **(F)** treated with PBS or Ang II for 48 h (*n* = 3 independent experiments; ^*^P < 0.05 vs. AdshRNA/PBS or vs. AdGFP/PBS; ^#^*P* < 0.05 vs. AdshRNA/Ang II or vs. AdGFP/Ang II). All the data are presented as the mean ± SD.

To understand the functional role of TANK in cardiomyocytes, AdshTANK was infected to knockdown TANK. Also, AdTANK is used to overexpress TANK. the cells were then incubation with 1 μM Ang II or PBS for 48 h and immunostained with α-actin. First, the effectiveness of knockdown or overexpression of TANK in cardiomyocytes was confirmed (Figure 2A). As shown in Figures 2B,C, cardiomyocyte hypertrophy was significantly inhibited the AdshTANK group incubated with 1 μM Ang II for 48 h compared with the AdshRNA group. In contrast, the cell size of the AdTANK group was markedly increased under the stimulation of Ang II, compared to the AdGFP group (Figure 2D). Similarly, the mRNA levels of ANP and β-MHC decreased in the TANK-knockdown group, while the levels were upregulated after TANK overexpression, which supports the observations from cell morphology (Figures 2E,F). These data confirm that TANK is a positive regulator of cardiomyocyte hypertrophy. In addition, we found similar result in TANK-TG mice when infused with Ang II (Supplementary Figure 2).

### TANK Cardiomyocyte-Specific Deficiency Alleviates Hemodynamic Overload-Induced Cardiac Hypertrophy

**Figure 3:**
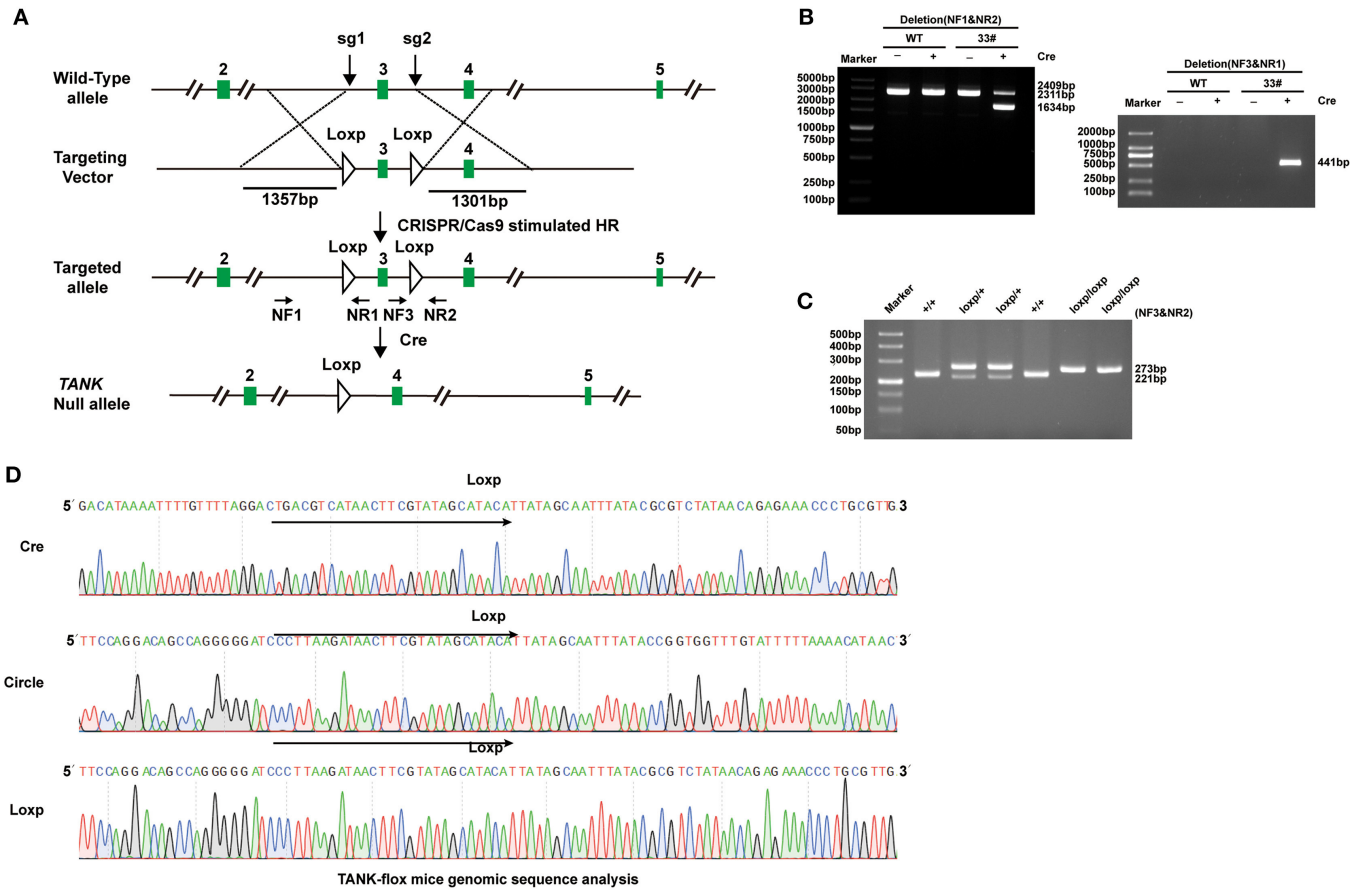
Establishment of TANK-CKO mice. **(A)** Schematic diagram of the construction of cardiac-specific TANK knockout mice. **(B)** PCR and 3% gel electrophoresis were used to identify the recombinant TANK-gene Floxed site in founder mice mediated by Cre-loxP. Genomic DNA from founder mouse (33#) was processed using Cre recombinant enzyme and used as a template. NF1 & NR2 primers and NF3 & NR1 primers are respectively used for amplification and then gel electrophoresis was performed. A smaller band appeared in the NF1 & NR2 amplification (1634 bp), NF3&NR1, which originally extended in the opposite direction, amplified a bright band. Among all bands, 2409bp band was loxP Founder mouse genome, 2311bp band was WT mouse genome, and 441bp band was cyclization PCR product. **(C)** PCR and 3% gel electrophoresis were used to identify TANK-flox alleles, in which the single 273bp band represented homozygous insertion of loxP biallele (loxP/loxP);221bp/273bp bands represented heterozygous insertion of loxP single allele (loxP /+); and the single 221bp band represented WT alleles (+/+). **(D)** Sequencing results of truncated PCR products and cycled PCR products in **(B)** and TANK-flox allele in **(C)**.

**Figure 4:**
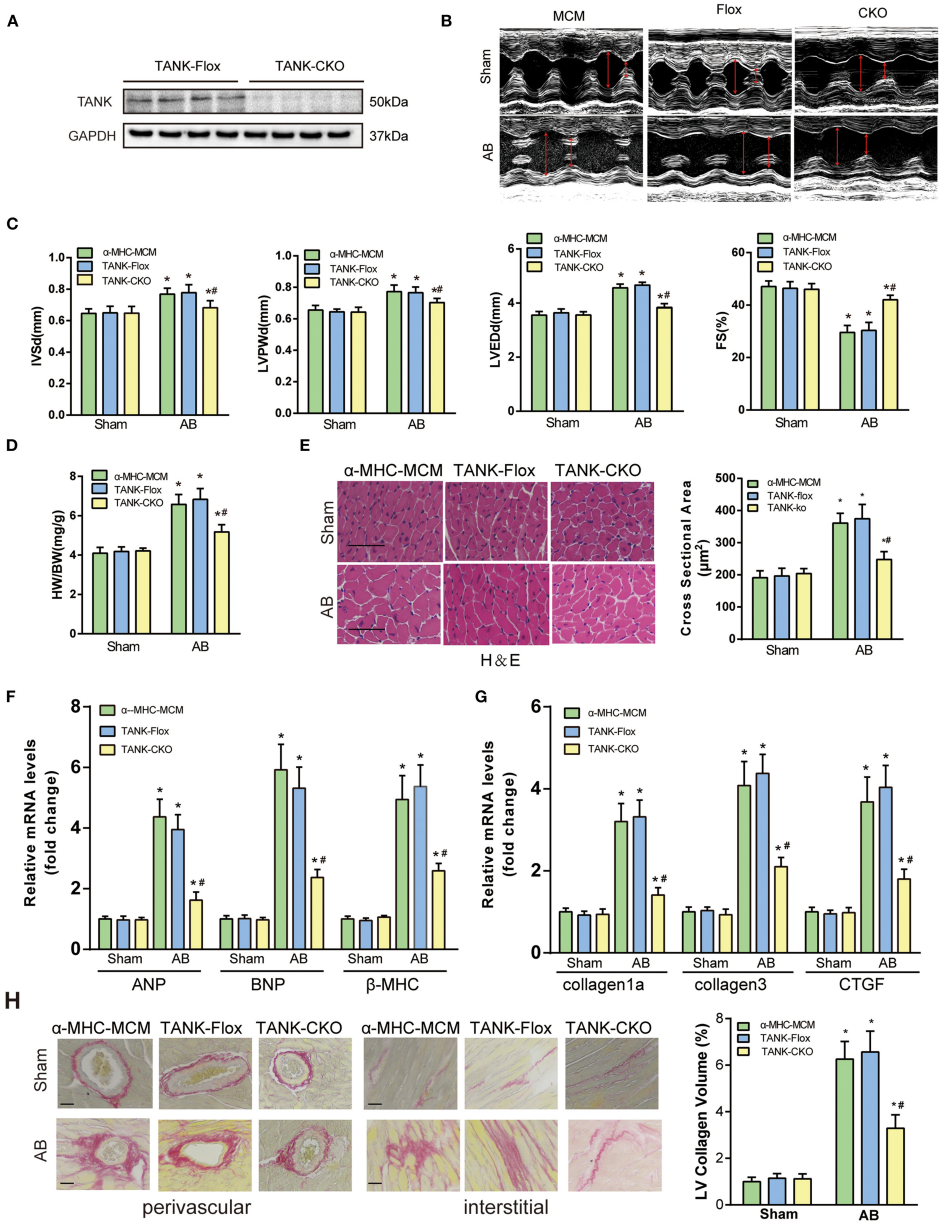
TANK cardiac-specific deficiency ameliorates pressure overload-induced cardiac hypertrophy. **(A)** Representative western blot showing TANK expression in the TANK-Flox and TANK conditional knockout (TANK-CKO) groups (*n* = 4 mice per group). **(B)** M-mode echocardiograms from control groups and TANK-CKO mice after AB. **(C)** End-diastolic interventricular septum (IVSd), posterior wall dimensions (LVPWd), left ventricular end-diastolic diameter (LVEDd) and fractional shortening (FS%) are measured by echocardiography from α-MHC-MCM, TANK-Flox, and TANK-CKO mice (*n* = 12-14 mice per group). **(D)** Ratio of heart weight (HW)/body weight (BW) in the control groups (α-MHC-MCM, TANK-Flox) and TANK-CKO group subjected to sham or aortic banding for four weeks (*n* = 12-14 mice per group). **(E)** Left, Histological analysis of hematoxylin and eosin (H&E) staining in α-MHC-MCM, TANK-Flox, and TANK-CKO mice at four weeks after sham operation or aortic banding surgery (*n* = 6 mice per group). Scale bars, 50 μm. Right, Quantification of the myocyte cross-sectional area of the indicated group (*n* = 100+ cells per each experimental group). **(F)** mRNA expression of the hypertrophic markers ANP, BNP, and β-MHC in α-MHC-MCM, TANK-Flox, and TANK-CKO mice four weeks after the sham operation or aortic banding surgery (*n* = 4 mice per group). **(G)** mRNA levels of the fibrotic markers collagen Iα, collagen III, and connective tissue growth factor (CTGF) in α-MHC-MCM, TANK-Flox, and TANK-CKO mice four weeks after sham operation or aortic banding surgery (*n* = 4 mice per group). **(H)** Left, representative image of picrosirius red staining to detect fibrosis in α-MHC-MCM, TANK-Flox, and TANK-CKO mice four weeks after sham operation or aortic banding surgery (*n* = 6 mice per group); scale bars, 50 μm. Right, quantification of left ventricle (LV) collagen volume (*n* ≥ 40 fields per group). All the data are presented as the mean ± SD. *P < 0.05 vs. α-MHC-MCM/Sham or TANK-Flox/Sham or TANK-CKO/Sham; ^#^*P* < 0.05 vs. TANK-Flox/AB or α-MHC-MCM/AB.

To further clarify the potential role of TANK in the development of cardiac hypertrophy, a mouse model of TANK-CKO was generated (Figures 3A–D). TANK expression was detected and we found remarkable reduction in TANK-CKO mice compare with that in TANK-Flox mice (Figure 4A). M-mode echocardiograms from each group were as shown in Figure 4B. At baseline, there was no difference in phenotypic characteristics among groups. After AB, cardiac function was evaluated by echocardiogram and we found that the IVSd, LVPWd, and LVEDd of TANK-CKO mice were markedly decreased, and FS% increased compared with those in the control groups (Figure 4C). Heart weight/bodyweight (HW/BW) ratio showed a sharper decline in TANK-CKO mice than in the control groups 4 weeks (Figure 4D). Histological examination of the heart showed that the size of cardiomyocytes from TANK-CKO mice was decreased compared to that in the control mice after 4 weeks of aortic banding (Figure 4E). Consistently, expression levels of hypertrophic markers as mRNA levels of ANP, BNP, and β-MHC were decreased in TANK-CKO mice (Figure 4F). Cardiac fibrosis was assessed and it was found that content of collagen in interstitial and perivascular space was significant reduced in TANK-CKO mice, compared with that in control groups after AB surgery. mRNA levels of the fibrotic markers in TANK-CKO mice also decreased, including collagen Iα, collagen III, and connective tissue growth factor (CTGF) (Figures 4G,H). These data suggest that TANK deficiency exerts a protective effect on cardiac hypertrophy.

### TANK Overexpression Results in the Exacerbation of Hemodynamic Overload-Induced Cardiac Remodeling

**Figure 5:**
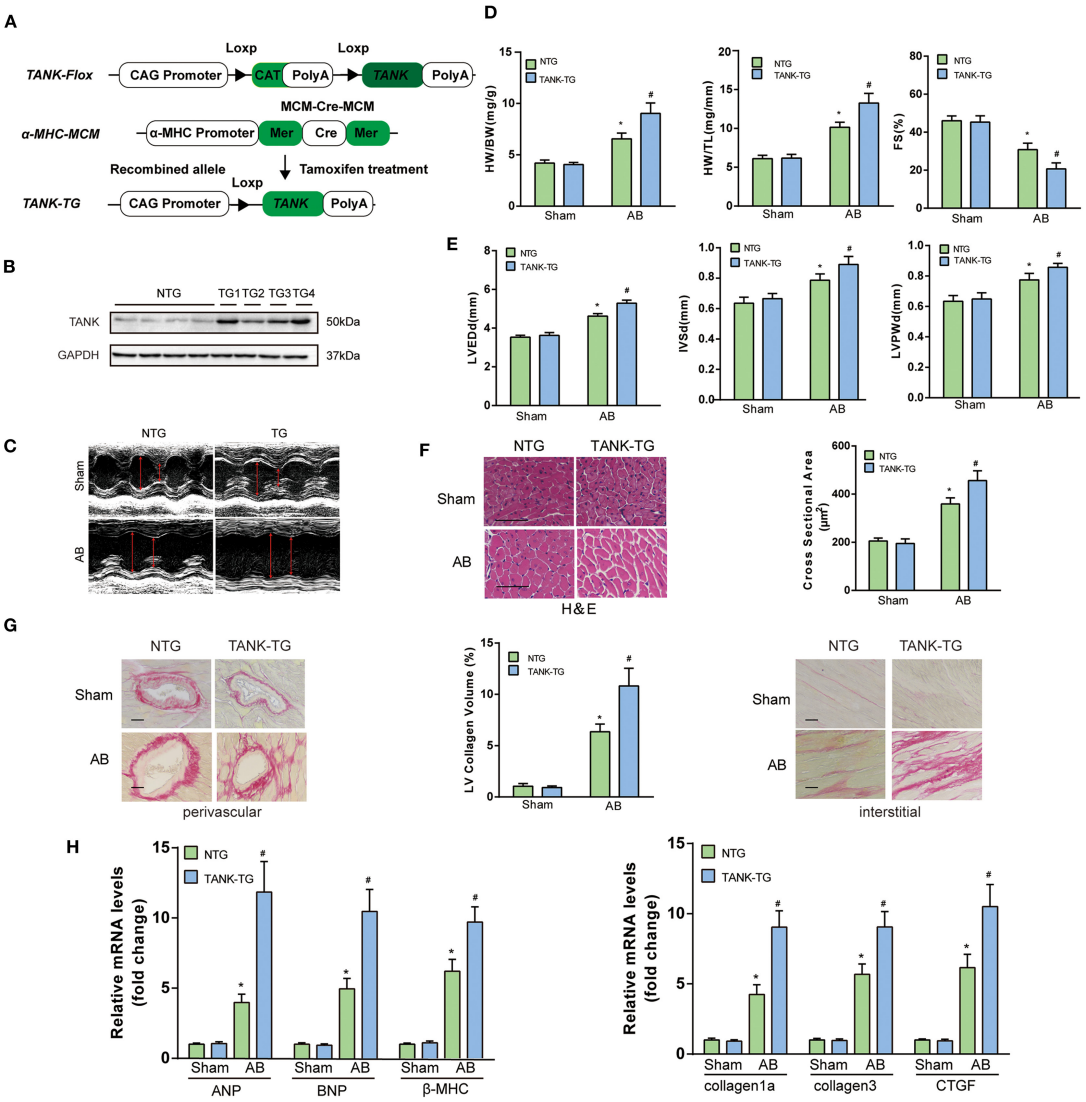
TANK cardiac-specific overexpression results in the exacerbation of pressure overload-induced cardiac hypertrophy. **(A)** Schematic diagram of the construction of cardiac-specific TANK overexpression experimental mice. **(B)** Western blot showing TANK expression in mouse heart samples from nontransgenic (NTG) mouse hearts and cardiac-specific TANK transgenic (TANK-TG) hearts (TG1, TG2, TG3, and TG4; *n* = 4 mice per group). **(C)** M-mode echocardiograms from NTG group and TANK-TG mice after AB. **(D)** The HW/BW and HW/TL ratio determined in NTG and TANK-TG mice subjected to sham or AB treatment for four weeks (*n* = 12-14 mice per group). **(D,E)** M-mode echocardiograms of NTG mice and TANK-TG mice after AB. Measurements of LVEDd, IVSd, LVPWd, and FS% using echocardiography of NTG and TANK-TG mice subjected to sham or AB treatment for four weeks (*n* = 12–14 mice per group). **(F)** Representative histological cross-sections stained with H&E indicated concentric hypertrophy in NTG and TANK-TG mice subjected to four weeks of AB treatment (*n* = 6 mice per group) (Left). Cross-sectional area of the indicated group was quantified (*n* = 100+ cells per group) (Right). **(G)** Picrosirius red staining showing perivascular fibrosis (Left) and interstitial fibrosis (Right) in heart sections of NTG and TANK-TG mice (*n* = 6 mice per group) , Scale bars, 50 μm. Middle, Statistical results of LV collagen volume of the indicated group (*n* ≥ 40 fields per group). **(H)** The relative mRNA levels of the hypertrophic markers ANP, BNP, and β- MHC (Left), or fibrotic markers collagen Iα, collagen III, and CTGF (Right) in NTG and TANK-TG mice (*n* = 4 mice per group) subjected to sham or AB treatment for four weeks. All the data are presented as the mean ± SD. ^*^P < 0.05 vs. NTG/Sham or TANK-TG/Sham; ^#^*P* < 0.05 vs. NTG/AB.

TANK-TG mice were established (Figure 5A) and the expression in different tissue was confirmed using Western blotting analysis (Figure 1E). The line showing the highest expression was chosen as the experimental animal group (Figure 5B). There was no significant distinction in morphology or pathology of the heart between TANK-TG and NTG mice. M-mode echocardiograms from NTG mice and TANK-TG mice after AB were shown in Figure 5C. After 4 weeks subjected to AB surgery, the TANK-TG mice exhibited higher ratios of HW/BW and HW/TL than NTG mice (Figure 5D). To determine if TANK-TG was associated with heart dysfunction, echocardiograms were performed. As shown in Figures 5D,E, TANK-TG showed an increase in LVEDd, IVSd, and LVPWd, and a decrease in FS%. The cross-sectional area and cardiomyocyte size were also analyzed by HE staining and showed a significant increase in TANK-TG mice relative to NTG mice after AB surgery (Figure 5F). Similarly, the overexpression of TANK resulted in up-regulation of collagen content (Figure 5G). Consistently, higher mRNA expression levels of hypertrophic markers were detected in TANK-TG mice, including ANP, BNP, and β-MHC; fibrosis-related markers were also elevated, such as collagen Iα, collagen III, and CTGF (Figure 5H). Taken together, these data demonstrate that cardiomyocyte-specific TANK overexpression aggravates the pressure overload-induced hypertrophic response.

### TANK Promotes Cardiac Hypertrophy by Activating AKT Phosphorylation and Leads to Fibrosis Under Control of TGF-β1 Signaling Pathway

**Figure 6:**
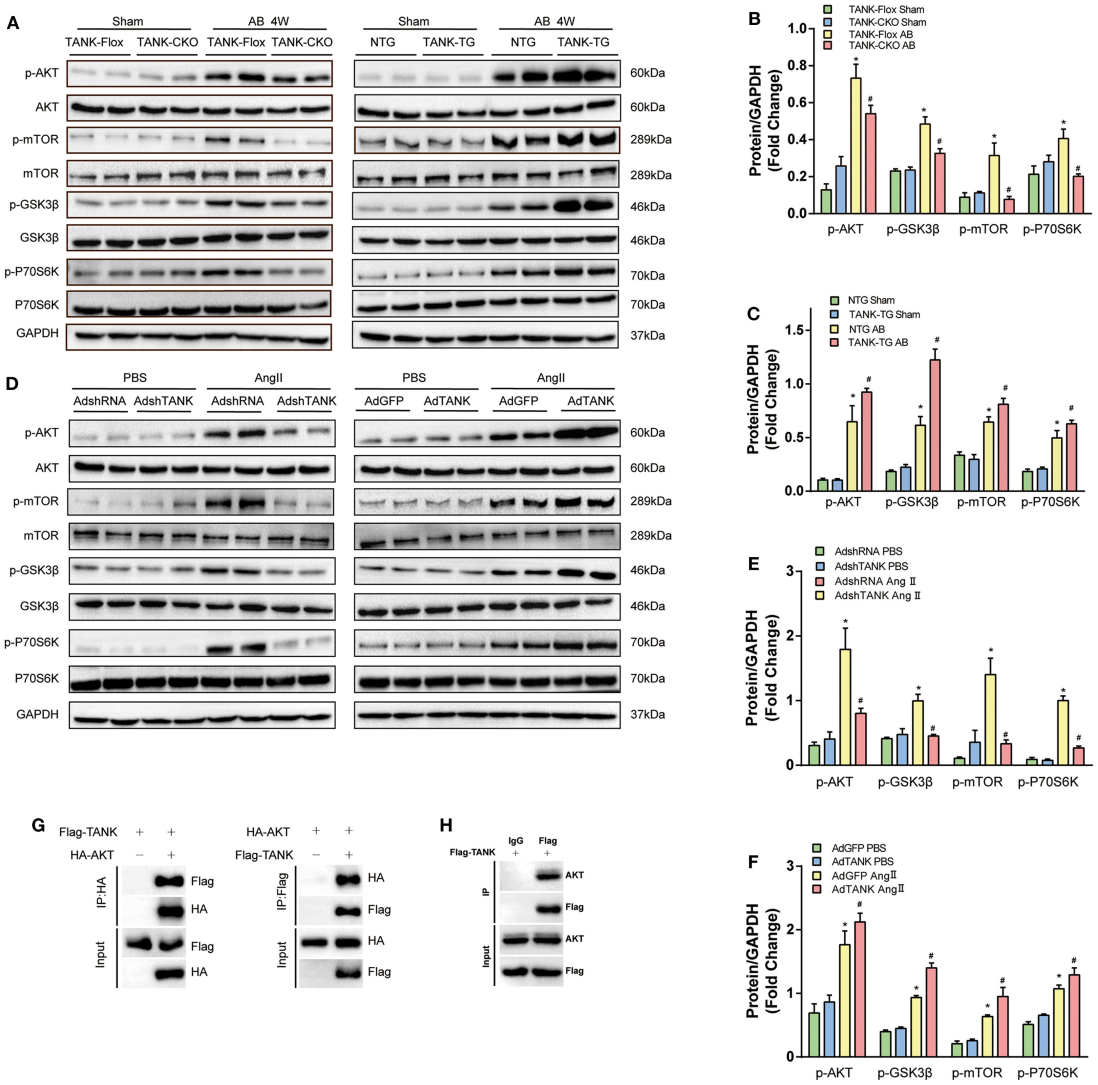
TANK promotes AKT phosphorylation *in vivo* and *in vitro*. **(A)** Representative Western blot showing total and phosphorylated expression of AKT, GSK3β, mTOR, and P70S6K in TANK-CKO mice compared with TANK-Flox mice (Left) or TANK-TG mice compared to TANK-NTG mice (Right) subjected to sham or aortic banding for four weeks. **(B ,C)** Quantitative analysis of the phosphorylation of AKT, GSK3β, mTOR, and P70S6K in TANK-Flox and TANK-CKO mice **(B)** or in TANK-NTG and TANK-TG mice **(C)** (*n* = 4 per group; ^*^P < 0.05 vs. TANK-Flox/Sham or TANK-NTG/Sham; ^#^*P* < .0.05 vs. TANK-Flox/AB or TANK-NTG/AB). **(D)** Representative Western blot for the AKT signaling-related protein from NRCMs infected with AdshRNA as the control and AdshTANK to delete TANK expression (Left) or AdGFP as the control and the AdTANK group to overexpress TANK protein (Right) treated with PBS or Ang II. **(E,F)** Quantitative analysis of phosphorylated AKT signaling-related protein in the AdshRNA and AdshTANK groups **(E)** or the AdGFP and AdTANK groups **(F)** (*n* = 3 independent experimens; ^*^P < 0.05 vs. AdshTANK/PBS or AdGFP/PBS; ^#^*P* < 0.05 vs. AdshTANK/Ang II or AdGFP/Ang II). The data are presented as the mean ± SD. **(G,H)** Immunoprecipitation followed by immunoblotting revealed that TANK interact with TANK.

Since TANK is considered to promote cardiac hypertrophy, the underlying mechanism was investigated. A multitude of signaling pathways associated with hypertrophy are well-established, in which the MAPK and AKT pathways are thought to be the two most important pathways (19). First, we explored whether TANK activates MAPK signaling pathways. As shown in Supplementary Figure 1A, there was no obvious distinguishable activation of MEK1/2, ERK1/2, and p38 using Western Blot analysis between groups (CKO vs. Flox and TG vs. NTG). Next, the AKT signaling pathway was evaluated and it was found that TANK-deficient mice subjected to AB surgery experienced decreased AKT phosphorylation levels compared to Flox mice, while the activity of phosphorylated AKT levels was enhanced in TANK-TG mice after AB (Figures 6A–C). The total AKT level among all groups was not significantly different. The downstream molecules involved were detected in the same manner. In AB-treated TANK-CKO mice, the phosphorylation of mTOR and P70S6K was downregulated and phosphorylated GSK3β decreased. Conversely, TANK overexpression exhibited the opposite effect on the phosphorylation of mTOR, P70S6K, and GSK3β.

We also confirmed activation of TANK on the AKT signaling pathway in neonatal rat cardiomyocytes (Figures 6D–F). Analysis using Western blotting revealed that the phosphorylation of AKT/mTOR/P70S6K induced by Ang II dramatically declined in Ad-shTANK cells but markedly increased in Ad-TANK cells. These results indicate that TANK leads to hypertrophy, most likely through mediation of the AKT signaling pathway.

Furthermore, Immunoprecipitation was performed and demonstrated that TANK is able to interact with AKT (Figures 6G,H).

In addition, the underlying mechanism of interstitial fibrosis were also investigated using WB. As TGF-β1 is crucial for cardiac fibrosis, the expression of TGF-β1 and related molecular were detected in TANK-CKO mice and TANK-TG mice. After AB, we noted that TANK-CKO hearts had decreased amount of TGF-β1 and phosphorylated Smad2 and Smad3 (Supplementary Figure 1B). Besides, TANK-TG hearts show increased expression of these protein after AB compared with NTG hearts.

### Blockage of AKT Signaling Reverses Cardiac Hypertrophy due to TANK Overexpression

**Figure 7:**
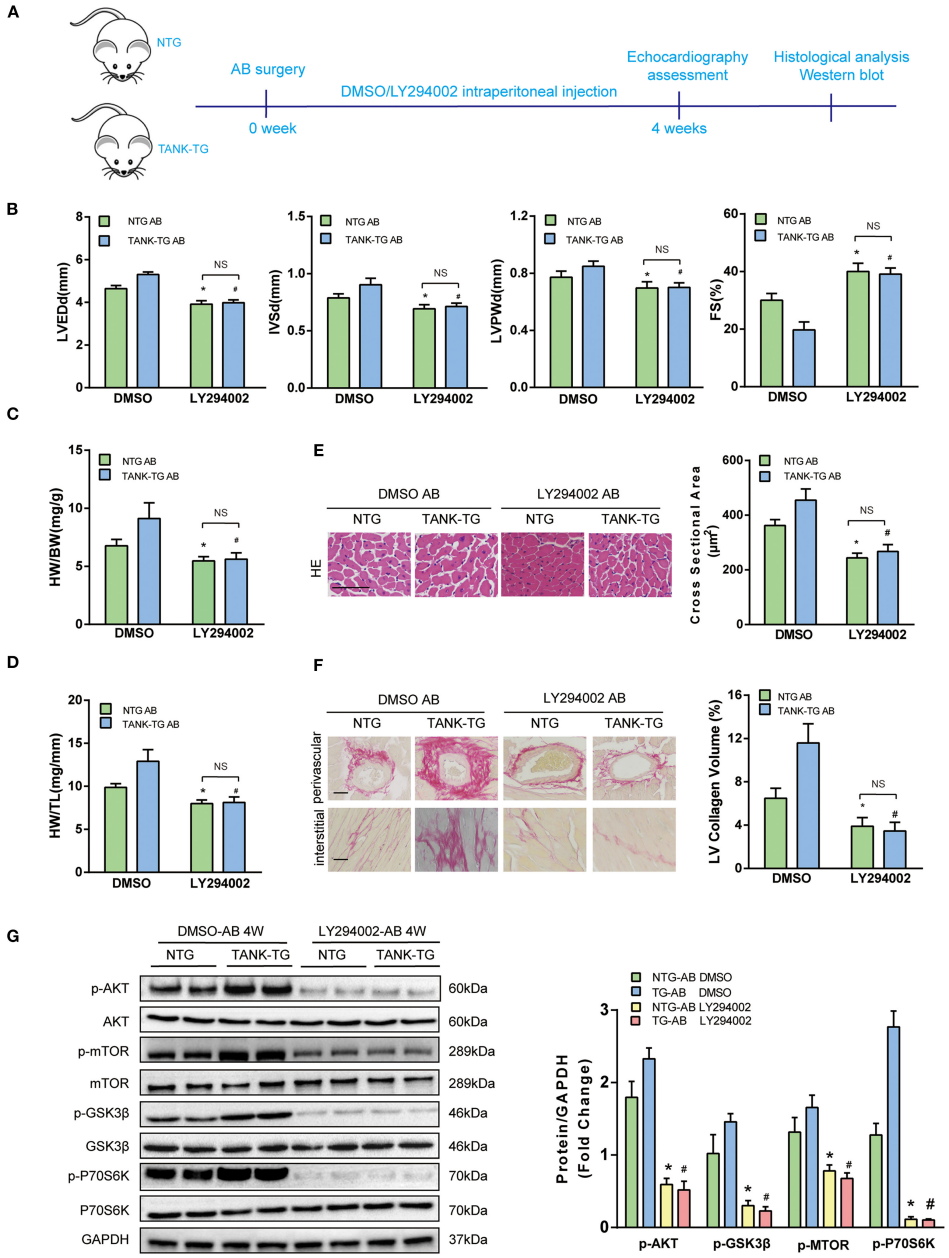
Effect of PI3K inhibitor LY294002 on cardiac hypertrophy in TANK-TG mice. **(A)** A flowchart to illustrate animal experiments. **(B)** Echographic parameters obtained from heart samples of the NTG and TANK-TG mice injected with DMSO or LY294002 after aortic banding for four weeks (*n* = 4 mice per group). **(C,D)** Quantitation of HW/BW and HW/HL ratios of NTG and TANK-TG after four weeks aortic constriction and treatment with DMSO or LY294002 (*n* = 12 mice per group). **(E)** Left, H&E stained heart sections of NTG and TANK-TG mice after four weeks of aortic constriction and treatment with DMSO or LY294002 (*n* = 6 mice per group) ,Scale bars 50 μm. Right, quantification of the cross-sectional area of the indicated group (*n* ≥ 100 cells each experimental group). **(F)** Left, PSR-stained heart sections of DMSO- and LY294002-treated NTG and TANK-TG mice with four weeks of aortic banding (*n* = 6 mice per group), Scale bars 50 μm. Right, quantification of the LV collagen volume of the indicated group (*n* ≥ 40 fields per each experimental group). **(G)** Left, Western blots showing the effect of LY294002 on the phosphorylation of AKT and its substrates of NTG and TANK-TG mice after four weeks of aortic constriction. Right, quantitation of Western blot bands. All data are presented as the mean ± SD (*n* = 4 per group;^*^P < 0.05 vs. NTG/DMSO AB; #*P* < 0.05 vs. or TANK-TG/DMSO AB).

**Figure 8:**
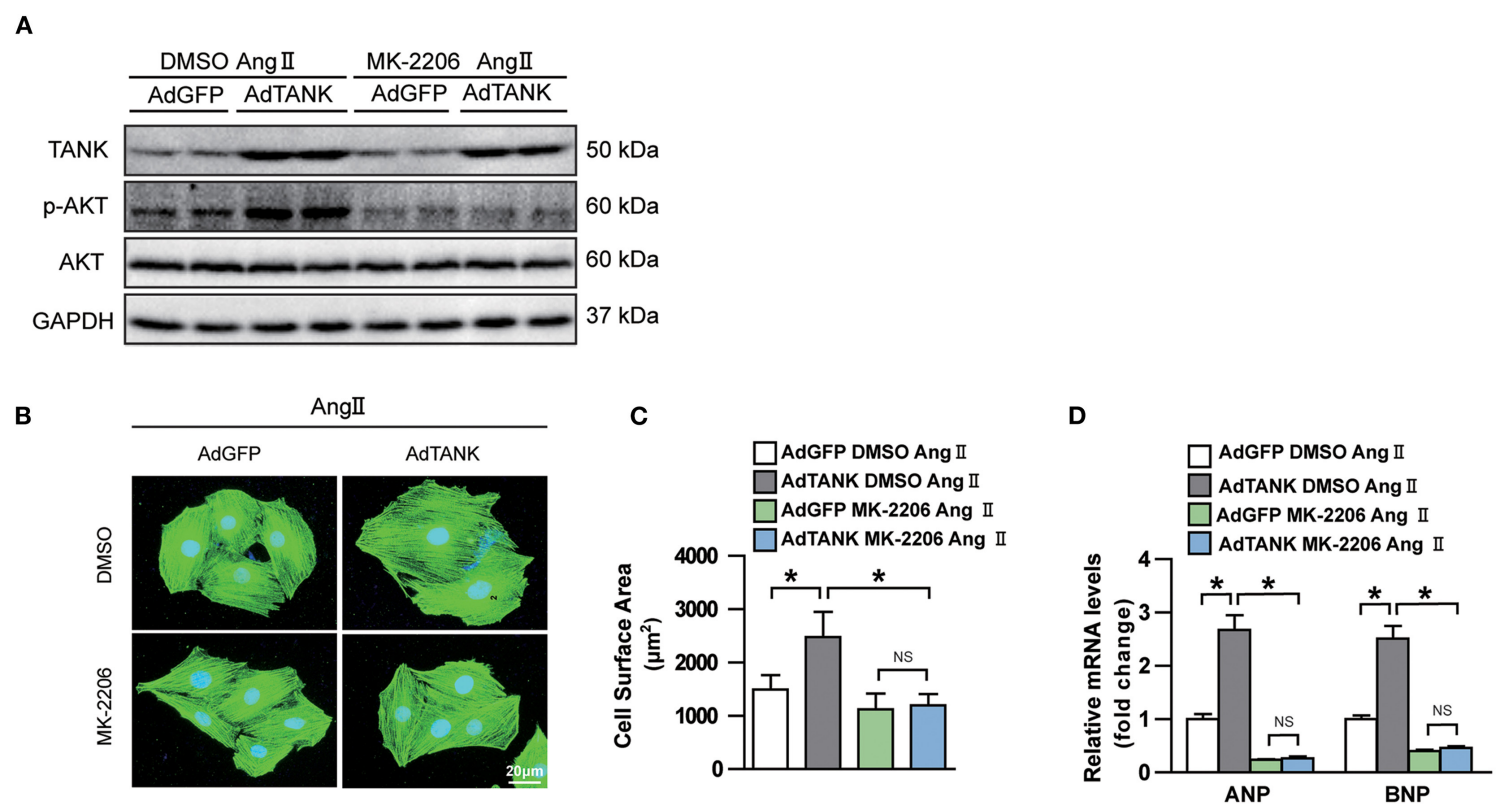
Effect of AKT inhibitor MK-2206 on AngII-induced cardiomyocyte hypertrophy in TANK overexpression NRCMs. **(A)** Western blot showing MK-2206 inhibits phosphorylation of AKT after stimulation of AngII. **(B)** NRCMs infected with the indicated adenoviruses are incubated with MK-2206 or DMSO after stimulated with AngII. Sarcomere organization was stained using anti-α-actinin antibodies (green), and nuclei were determined by DAPI staining (blue). scale bar, 20 μm. **(C)** Quantification of cell surface area of indicated groups after MK-2206 or DMSO treatment (*n* > 50 cells per experimental group). **(D)** mRNA level of hypertrophic marker ANP and BNP in TANK overexpression cell and control group treated with MK-2206 or DMSO after stimulated with AngII (*n* = 3 independent experiments). ^*^P < 0.05.

Having shown that TANK promotes the activation of the AKT signaling pathway under hemodynamic overload condition, blockage of the AKT signaling pathway was performed to identify whether it would reverse cardiac hypertrophy. Finally, a pharmacological inhibition strategy was performed in TANK-TG mice with PI3K inhibitor LY29004 and AKT inhibitor MK2206. Figure 7A shows an animal experimental flowchart. LY29004-treated mice displayed less increased LVEDd, IVSd, and LVPWd as well as preserved FS% compared to mice treated with DMSO (Figure 7B). Similarly, TANK-TG mice treated with LY294002 exhibited decreased HW/BW and HW/TL ratios 4 weeks after aortic banding, compared to DMSO-treated mice (Figures 7C,D). In addition, LY29004 significantly prevented against aortic banding-induced cardiac hypertrophy and fibrosis under the condition of TANK overexpression (Figures 7E,F). The AKT signaling-related molecules were also detected via Western blotting and are shown in Figure 7G. *In vitro* experiment, NRCMs overexpressed TANK exhibited larger cell size after Ang II stimulation accompanied by elevated hypertrophic makers ANP and BNP. Incubation with MK2206 can reverse myocyte hypertrophy induced by Ang II (Figure 8). Therefore, we demonstrated that TANK may hasten hypertrophy through the AKT signaling pathway.

## Discussion

TANK is a scaffold protein that binds to at least two other signaling proteins and lacks enzymatic activities (20). Scaffold proteins can function as platforms to organize signaling molecules into functional complexes, locate signaling molecules at particular sites in cells, integrate feedback signals, and prevent activation signaling molecules from being deactivated. Previous studies have noted emergence of scaffold proteins as important modifiers in the regulation of cardiac hypertrophy. IQGAP1 (IQ motif-containing GTP-ase protein (1) is key to c-Raf-MEK1/2-ERK1/2 as well as AKT signaling, and regulates pathological cardiac remodeling upon pressure overload (21). FHL1 (four-and-a-half LIM domains (1) senses biomechanical stress and promotes cardiac hypertrophy by affecting the MAPK signaling cascade (22). ANKRD (Ankyrin repeat domain1), a sarcomere scaffolding protein, induces cardiac hypertrophy by increasing the phosphorylation of ERK-GATA4 after phenylephrine (PE) stimulation (23). In this study, for the first time, to the best of our knowledge, we identified TANK as a scaffold protein activate AKT signaling in pathological cardiac hypertrophy, and provide future evidence for the IKKε-TBK1/AKT signaling pathway.

The importance of TANK in both the innate immune response and non-infectious inflammation has been observed in previous studies. TANK deficiency dampens type I interferon gene induction and enhances cell susceptibility to multiple viruses (11, 24). Besides, TANK was proven as a novel target of one type of viral protease of RNA virus named 3C protease. Encephalomyocarditis Virus 3C protease cleaved TANK not only enhanced TRAF6-induced NFκB signaling but also disrupted TANK-TBK1-IKKε-IRF3 complex, leading to a significant reduction of IFN production, and evaded the host innate immune responses (25, 26). Similarly, Seneca Valley virus (SVV) cleaved TANK via 3C protease promotes TRAF6 mediated NFκB activation and suppression of IFN mediated inflammation (27). In addition, the deletion of TANK suppressed the development of fatal glomerulonephritis caused by intestinal commensal microflora (28). In renal ischemia-reperfusion injury, the expression of TANK is also persistently upregulated, but its functional contribution has not yet been confirmed (29). Moreover, TANK plays a critical role in glioblastomas as an activator in S-phase progression and cell migration (19). Emerging evidence indicates that the expression of TANK is ubiquitously detected in various tissues, including heart tissues (29), but the expression levels of TANK under prohypertrophic stimuli remain unclear. Here, we found that TANK expression was markedly elevated in heart samples of mice subjected to aortic banding compared with that in Sham hearts. Similarly, TANK expression was progressively upregulated in NRCMs incubated with ANG II. The regulation mechanism of TANK expression has not been fully clarified. Transcription factor SOX11, a member of the SoxC family, is essential for the development of the cardiac outflow tract (30). However, there is no evidence that it is involved in the progression of cardiac hypertrophy. NFκB is considered another important modulator for TANK expression. The TNF-α signal triggers the p50-p65 heterodimer to translocate into the nucleus, and induces the expression of TANK (31). However, the increased expression of TANK during cardiac hypertrophy requires further research.

To explore the underlying mechanism of TANK involved in pathological pressure overload-induced cardiac hypertrophy, conditional transgenic mice were utilized in combination with aortic constriction, which is an effective approach for the study of hypertrophy *in vivo*. The results of this study indicate that TANK is functionally important during press overload, as TANK-CKO mice exhibit thinner ventricular walls, left chamber dilation, alleviative contractile dysfunction, and reduced reactivation of cardiac fetal genes when exposed to persistent aortic constriction. Another important detection in TANK-CKO mice is reduced fibrosis, which is a typical feature of pathologic cardiac hypertrophy (32). Compared with NTG mice, transgenic mice overexpressing TANK present exaggerated cardiomyocyte hypertrophy and interstitial fibrosis. These data provide direct evidence that TANK is an pathological hypertrophy accelerator.

Pathological hypertrophy caused by changes in signal transduction pathways responding to a series of stimuli has established MAPKs as classical proteins that are critical for cardiac hypertrophy (33, 34). Herein, we demonstrate that the altered TANK expression has no effect on MAPK signaling in the myocardium but increases the phosphorylation of AKT as well as activation of mTOR and S6K, and IP analysis revealed TANK interacts with AKT physically. AKT participates in cardiac hypertrophy ranging from cell survival to aging. Insulin-like growth factor 1 and exercise can lead to AKT phosphorylation and eventually cause physiological adaptive cardiac hypertrophy (35). However, additional experiments showed that the constitutive cardiac-specific overexpression of AKT1 cause elevated heart weight and pathological hypertrophy-associated enlarged cell size, impaired contractile function, and interstitial fibrosis (36, 37). AKT3, which is functionally distinct from AKT1 in different cell types, also play a role in diseased human hearts. AKT3 transgenic mice exhibit pathological hypertrophy at 20 weeks of age (38). However, a different observation showed that AKT1-deficient mice result experience increased susceptibility to hypertrophic stimuli and more profound cardiac hypertrophy in response to aortic constriction (39). Phosphorylation is the most important post-translational determinant of AKT activity (40), PI3K is required for AKT membrane recruitment. We demonstrate that PI3K inhibitor LY294002 can reverse the phenotypic spectrum caused by aortic constriction, especially in TANK-TG mice and MK2206, a highly selective inhibitor of AKT, can reverse myocyte hypertrophy induced by Ang II.

As a Ser/Thr protein kinase, mTOR (mechanistic target of rapamycin) plays a critical role downstream of AKT. Once modulated, mTOR transduces signals to different effectors, such as P70S6K1, 4E-BP1, SREBP1, Lipin, and HIF1, and participates in protein synthesis and cell metabolism (41). mTOR is considered to be essential for pressure overload-induced pathological cardiac hypertrophy. Partial genetic deletion or pharmacological suppression of mTOR has been found to persistently ameliorate cardiac hypertrophy induced by AB (42). It is notable that mTOR activation alone is insufficient and requires coordination with other signaling pathways effectors to promote cardiac hypertrophy (43). Emerging evidence shows that epigenetic reprogramming participates in the contribution of mTOR during cardiac hypertrophy. The genetic and pharmacological downregulation of class I HDACs blunts pathological cardiac hypertrophy by inhibiting TSC2-dependent mTOR signaling (44). Chaer, a heart-enriched long non-coding RNA, interacts with PRC2 in a mTOR-dependent manner and inhibits histone H3 lysine 27 methylation at hypertrophic genes (45). Additionally, microRNA is a regulator of mTOR. MiR-99a suppresses aortic banding-induced cardiac hypertrophy targeting the mTOR/P70/S6K signaling pathway (46).

As far as we know, the interaction between TANK and AKT has not been reported before. N-terminus of TANK is essential for combination with ZC3H12A and TRAF6 (47). The Binding sites have been reported located in C-terminal and N-terminal domain of AKT. Previous lecture showed that downregulation of TANK impaired AKT phosphorylation (19). Interrupting TRIF-mediated complex formation composed of TRAF3, TANK, and IKKε leaded to downregulation of AKT phosphorylation, and eventually downregulation of inflammation (48). Also, TANK-binding kinase 1(TBK1), which form a ternary complex with TANK and TRAF2 (13), which activates AKT by direct phosphorylation (49, 50). Based on above information, we could deduce that TANK may directly or indirectly activated AKT by phosphorylation, therefore promote proliferation, inflammation etc.

Besides, cardiac fibrosis is regarded as a major factor leading to cardiac remolding and dysfunction. In our study, we also observed changes of cardiac fibrosis in transgenic mice models. TGF-β1 signaling pathway has been demonstrated correlated with cardiac fibrosis. Suppression of TGF-β1 signaling reduced cardiac fibrosis and prevent cardiac dysfunction in several models of cardiac remodeling (51). Therefore, the expression of TGF-β1 and related molecular were detected using western bolt in our experiment. After AB, TGF-β1 is upregulated in TANK-TG mice and induced increased fibrosis. Decreased TGF-β1 expression in TANK-CKO mice with pressure overload could alleviates cardiac fibrosis. According to previous studies, activation of TGF-β also could induce cardiomyocyte hypertrophy (52). In this term, inhibiting this signaling pathway may reverse the effects of TANK on cardiomyocyte hypertrophy. We found that AKT signaling pathway is involved in cardiac hypertrophy, blockage of AKT could reverse TANK overexpression induced hypertrophy. These two signaling pathways may cooperate in the process of TANK-related cardiac hypertrophy.

In latest studies, TANK was thought to respond to anti-TNF therapy in patients with autoimmune disease (53) and as a candidate gene associated with hepatitis C virus clearance in both African and European Americans (54). Our observation in this article might be a starting point for future clinical work on cardiac hypertrophy.

To the best of our knowledge, this study is the first to report TANK aggravates cardiac hypertrophy *in vitro* and *in vivo*. Moreover, we found that TANK could enhances the activation of AKT during pressure overload-induced pathological hypertrophy. With the ongoing development of new drugs, our findings have theoretical significance for the treatment of cardiac hypertrophy.

## Data Availability Statement

The original contributions presented in the study are included in the article/Supplementary Material, further inquiries can be directed to the corresponding author/s.

## Ethics Statement

The animal study was reviewed and approved by Shanghai Tongren Hospital Ethics Committee.

## Author Contributions

LJ conceived the study and provided financial support. YP, DW, and MM performed the experiment and collected the data. YP and XL wrote the paper. All authors contributed to the article and approved the submitted version.

## Conflict of Interest

The authors declare that the research was conducted in the absence of any commercial or financial relationships that could be construed as a potential conflict of interest.

## Publisher's Note

All claims expressed in this article are solely those of the authors and do not necessarily represent those of their affiliated organizations, or those of the publisher, the editors and the reviewers. Any product that may be evaluated in this article, or claim that may be made by its manufacturer, is not guaranteed or endorsed by the publisher.
