# Supplementary material for: TANK Promotes Pressure Overload Induced Cardiac Hypertrophy via Activating AKT Signaling Pathway
Source: Front Cardiovasc Med. 2021 Sep 3;8:687540. doi: 10.3389/fcvm.2021.687540 (PMC8446676; doi:10.3389/fcvm.2021.687540)
Supplement: Supplementary file 1 [file Data_Sheet_1.docx]

**Supplementary table 1**

**Primers for RT-PCR**

| Gene name | Forward primer (mouse) | Reverse primer (mouse) |
| --- | --- | --- |
| Anp | TCGGAGCCTACGAAGATCCA | TTCGGTACCGGAAGCTGTTG |
| Bnp | GAAGGACCAAGGCCTCACAA | TTCAGTGCGTTACAGCCCAA |
| β-Mhc | CAACCTGTCCAAGTTCCGCA | TACTCCTCATTCAGGCCCTTG |
| Collagen Iα | TGCTAACGTGGTTCGTGACCGT | ACATCTTGAGGTCGCGGCATGT |
| Collagen III | ACGTAAGCACTGGTGGACAG | CCGGCTGGAAAGAAGTCTGA |
| Ctgf | TGACCCCTGCGACCCACA | TACACCGACCCACCGAAGACACAG |
| Gapdh  TANK | ACTCCACTCACGGCAAATTC  GTCAAGTTTCCGCCTATGGA | TCTCCATGGTGGTGAAGACA  TATGGGGTCAATTCCCTGAA |

**Supplementary table 2**

**Primer information of Flag-TANK and HA-TANK**

| Gene name | Forward primer | Reverse primer |
| --- | --- | --- |
| *Flag-TANK* | CGAAGCCCGGGCGGATCCATGGAT  AAAAACATTGGCGAGCAAC | GGGCCCTCTAGACTCGAGTTAAGTCT  CTCCATTGAAGTGTGAATTAAG |
| *HA-AKT* | TCGGGTTTAAACGGATCCATGAGC GACGTGGCTATTGTG | GGGCCCTCTAGACTCGAGTCAGGCCG TGCCGCTG |
