# Supplementary material for: TANK Promotes Pressure Overload Induced Cardiac Hypertrophy via Activating AKT Signaling Pathway
Source: Front Cardiovasc Med. 2021 Sep 3;8:687540. doi: 10.3389/fcvm.2021.687540 (PMC8446676; doi:10.3389/fcvm.2021.687540)
Supplement: Supplementary file 2 [file Data_Sheet_2.doc]

**Caption of supplementary figures**

**Figure supplementary1 MAPK signaling pathway and TGF-β1 signaling pathway of control groups, TANK CKO mice and TANK-TG mice**

**(A)Left,** representative Western blots showing levels of total and phosphorylated MAPK signaling-related proteins in the hearts of sham- or AB-treated TANK-Flox and TANK-CKO mice (n=4 mice per group). **Right**, expression of total and phosphorylated MAPK signaling-related proteins in the hearts of sham- or AB-treated NTG and TANK-TG mice (n=4 mice per group). *P<0.05 vs TANK-Flox/Sham or TANK-NTG/Sham. **(B)Left,** total and phosphorylated TGF-β1 signaling pathway related proteins expression in the hearts of sham- or AB-treated TANK-Flox and TANK-CKO mice detected by western blot (n=4 mice per group). **Right**, western blot showing levels of total and phosphorylated TGF-β1 signaling pathway related proteins in the hearts of sham- or AB-treated NTG and TANK-TG mice (n=4 mice per group). *P<0.05 vs TANK-Flox/Sham or TANK-NTG/Sham; #P<0.05 vs TANK-CKO/Sham or TANK-NTG/Sham.

**Figure supplementary2 TANK overexpression promote AngII induced cardiac hypertrophy.**

**(A)**TANK expression in heart samples from non-transgenic mice and TANK-transgenic mice (n=4 mice per group). **(B-D)** HW/BW ratio, LW/BW ratio and HW/TL ratio of NTG mice and TANK-TG mice after treated with saline or AngII at 4 weeks (n=12-14 mice per group). **(E-G)** LVEDd, LVESd and FS% determined by echocardiography after AngII or saline injection after 4 weeks (n=12-14 mice per group). **(H)** Whole-heart sections stained with H&E showing aggravated hypertrophy in TANK transgenic hearts after AngII treatment compare with NTG mice (n=6 mice per group). **(I)** Fibrosis of indicated groups are detected by picrosirius red staining after AngII treatment (n=6 mice per group). *P<0.05
