# Supplementary figures and images for: TANK Promotes Pressure Overload Induced Cardiac Hypertrophy via Activating AKT Signaling Pathway
Source: Front Cardiovasc Med. 2021 Sep 3;8:687540. doi: 10.3389/fcvm.2021.687540 (PMC8446676; doi:10.3389/fcvm.2021.687540)

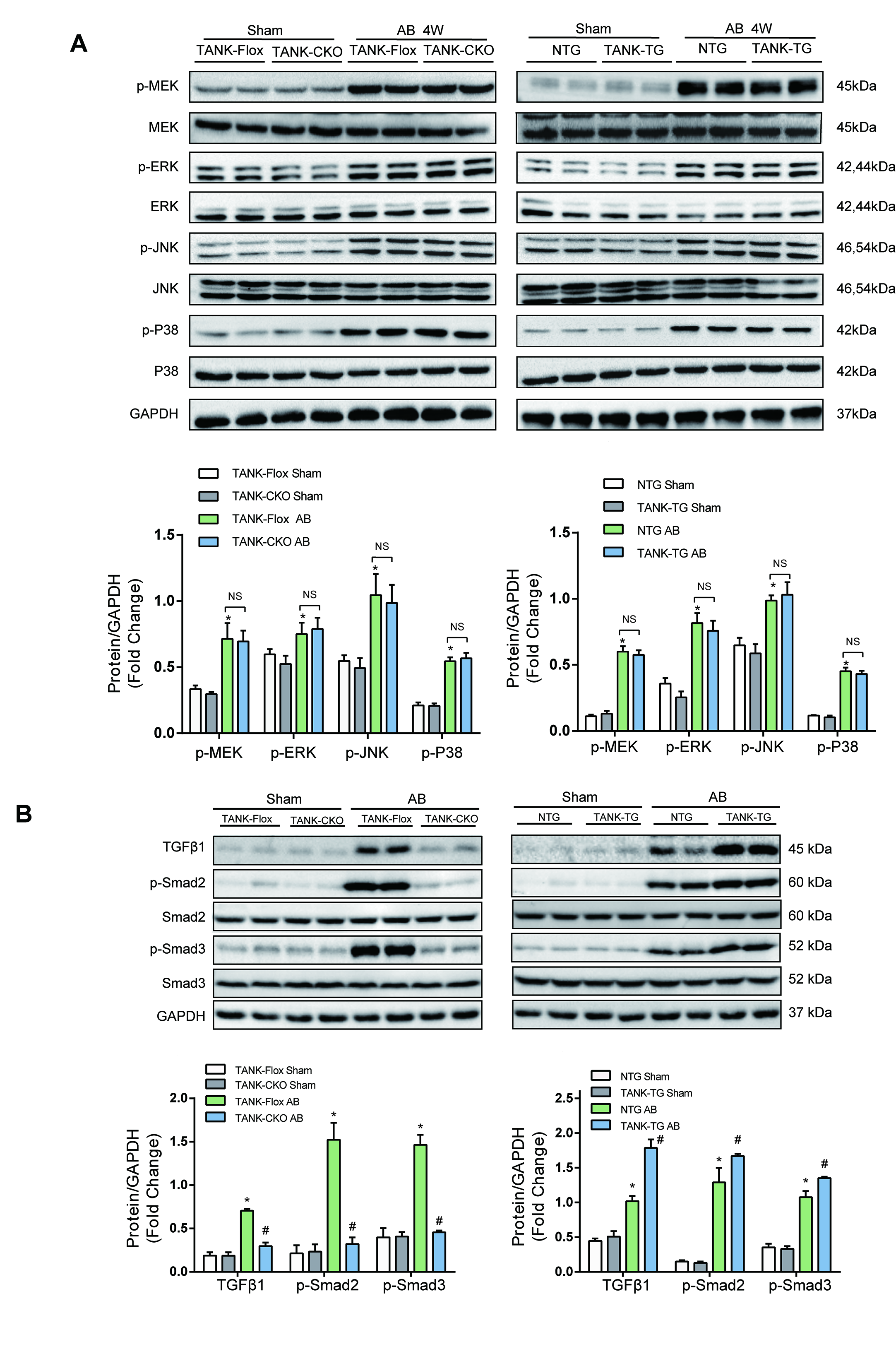

Supplement: Supplementary file 3 [file Image_1.TIF]

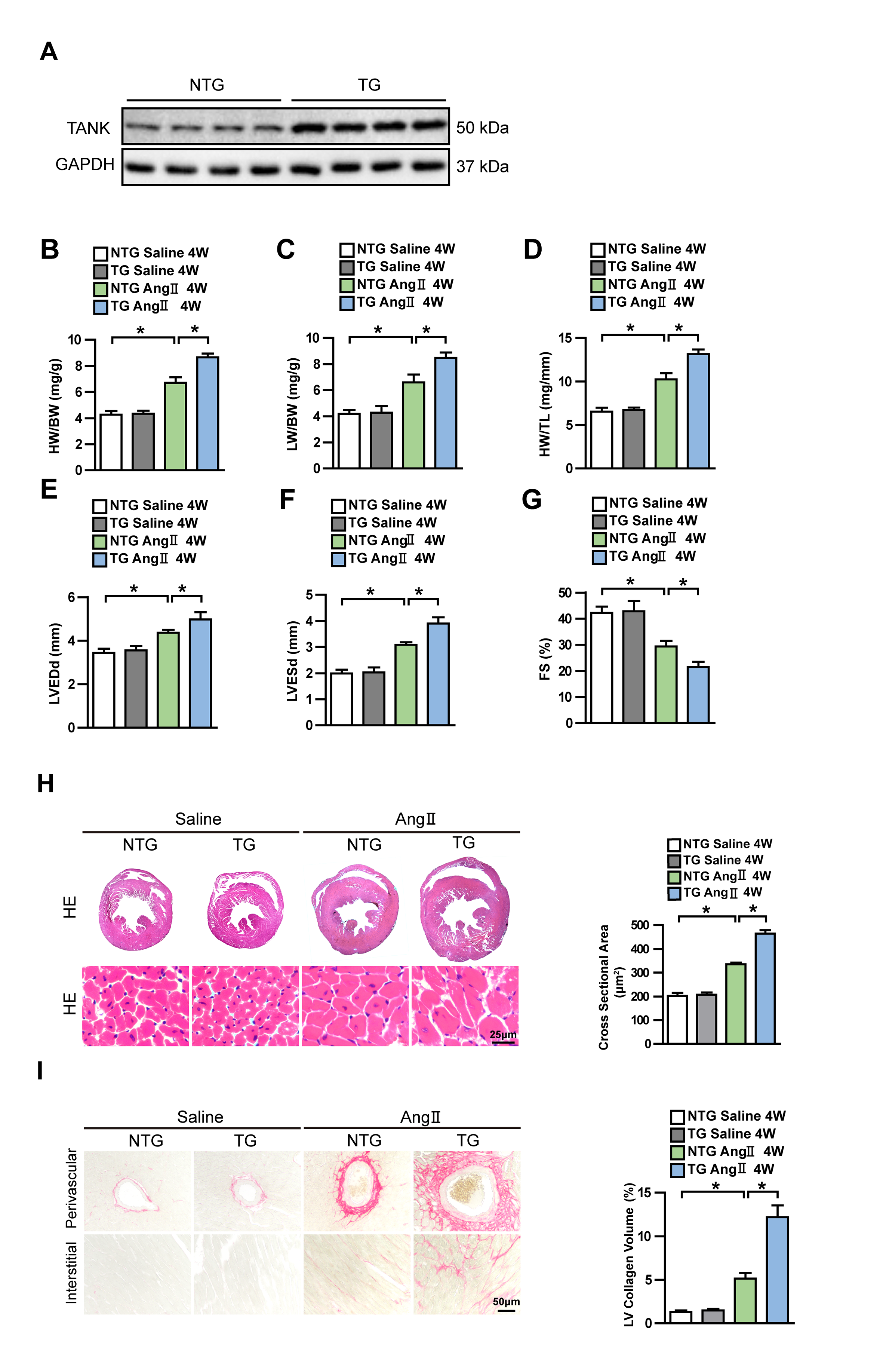

Supplement: Supplementary file 4 [file Image_2.TIF]
